# Two novel variants in *CEP152* caused Seckel syndrome 5 in a Chinese family

**DOI:** 10.3389/fgene.2022.1052915

**Published:** 2023-01-04

**Authors:** Li Zhang, Yanling Teng, Haoran Hu, Huimin Zhu, Juan Wen, Desheng Liang, Zhuo Li, Lingqian Wu

**Affiliations:** ^1^ Center for Medical Genetics, Hunan Key Laboratory of Medical Genetics, Hunan Key Laboratory of Animal Models for Human Diseases, School of Life Sciences, Central South University, Changsha, Hunan, China; ^2^ Hunan Jiahui Genetics Hospital, Changsha, Hunan, China

**Keywords:** Seckel syndrome, microcephaly, *CEP152*, whole-exome sequencing, mRNA splicing

## Abstract

**Background:** Seckel syndrome (SCKL) is a rare autosomal recessive inherited disorder, which is mainly characterized by intrauterine and postnatal growth restrictions, microcephaly, intellectual disability, and a typical “bird-head” facial appearance. Here, we aimed to identify the genetic etiology of a family with suspected SCKL.

**Methods:** This study enrolled a Chinese family suspected of SCKL with their detailed family history and clinical data. We performed karyotype analysis, copy number variation sequencing (CNV-seq), and trio whole-exome sequencing (WES) to explore the genetic etiology in the proband. Furthermore, the quantitative real-time polymerase chain reaction (PCR) and reverse transcription-PCR (RT-PCR) were conducted to confirm the pathogenicity of novel variants.

**Results:** The karyotype analysis and CNV-seq were normal in the proband. Two novel variants in *CEP152*, c.1060C>T (p.Arg354*) and c.1414-14A>G, were identified in the proband through trio-WES. The qPCR results showed that the total *CEP152* mRNA expression levels were significantly reduced in c.1060C>T (p.Arg354*) and c.1414-14A>G compared with healthy control individuals. Moreover, aberrant skipping of exon 12 due to the non-canonical splice-site variant was revealed by RT-PCR and Sanger sequencing.

**Conclusion:** Our findings expanded pathogenic variant spectra in SCKL and offered new insights into the pathogenicity of a non-classical splice-site variant in *CEP152*, which provided additional information for helping the family improve pregnancy plans in the future.

## 1 Introduction

Seckel syndrome (SCKL) is a rare autosomal recessive inherited disorder with high clinical heterogeneity and is mainly characterized by a typical “bird-head” facial appearance, severe microcephaly, intellectual disability, and a proportionate short stature (Seckel, 1960; Takikawa et al., 2008; Khojah et al., 2021). Other clinical symptoms, including low birth weight (Majewski and Goecke, 1982), cardiovascular anomalies (Khojah et al., 2021; Donmez et al., 2022), postnatal growth restriction (O’driscoll et al., 2004), and myelodysplasia (Verloes et al., 2013), were also occasionally reported.

So far, with the insight into the genetic underpinnings, this severe inherited disorder has been proved to be associated with pathogenic variants involved in DNA damage response pathways (O’driscoll et al., 2003), initiation of mitosis at cell cycle checkpoints (Casper et al., 2004), and centrosome duplication (Fu et al., 2016; Zheng et al., 2016). To date, nine classified subtypes of SCKL have been reported, according to different genetic causes, and at least 11 genes are responsible for the Seckel syndrome subtypes listed on Online Mendelian Inheritance in Man (OMIM).

Seckel syndrome 5 (MIM#613823, SCKL5) is caused by homozygous or compound heterozygous pathogenic variants in *CEP152*, located at 15q21.1 and contains 27 exons in total. *CEP152* codes a core protein of the centrosome with a molecular mass of 152 kDa, which is crucial in cell division. Meanwhile, it was proved to be involved in the maintenance of genomic integrity and is capable of responding to DNA damage (Andersen et al., 2003; Dinçer et al., 2017). Since the first identification of SCKL5 by Kalay et al. (2011) until now, only 13 cases have been reported worldwide. Hence, more patients and further studies are required to understand this rare disease better.

In this study, we described a 7-year-old boy with suspected Seckel syndrome. Further genetic testing, including karyotype analysis, copy number variation sequencing (CNV-seq), and whole-exome sequencing (WES), were performed to verify the diagnosis of this proband. All the tests and subsequent functional experiments revealed that novel compound heterozygous variants in *CEP152* would be the genetic cause for the phenotype observed.

## 2 Materials and methods

### 2.1 Subjects

**FIGURE 1:**
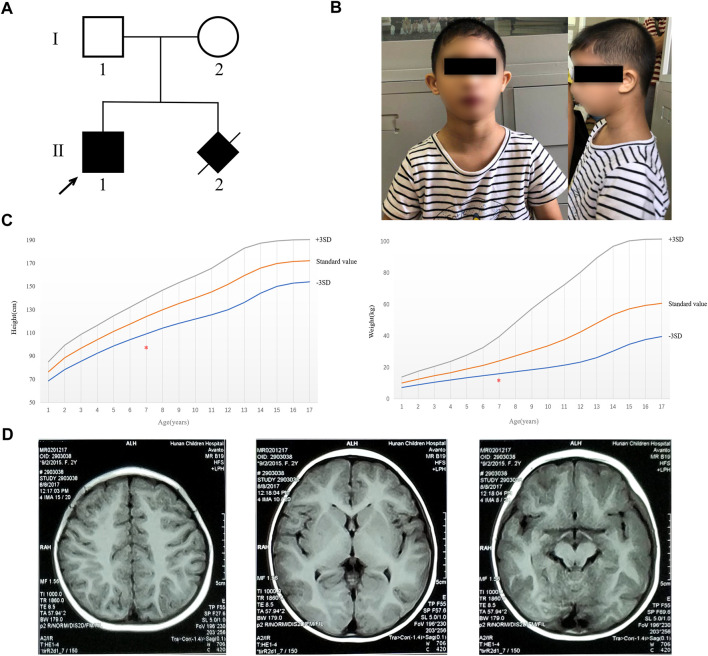
Pedigree of the family and clinical characterization of the proband. **(A)** Pedigree of the family. Filled black symbols indicate the affected subjects. The arrow indicates the proband in our study. **(B)** Clinical characteristics of the proband (including microcephaly, short stature, sloping forehead, high nasal bridge, beaked nose, and retrognathia). **(C)** Height and weight of the proband are marked with the red asterisk and were less than 3 SD of the standard value of people of the same age. **(D)** MRI result demonstrated that no abnormality was found.

The proband was a boy aged 7 years in 2018 from a non-consanguineous Chinese Han family (II-1, Figure 1A), who was mainly presented with microcephaly, short stature, intellectual disability, and dysphasia. His mother got pregnant again in 2013. The ultrasound showed that the fetus had intrauterine growth retardation, and they chose to terminate the pregnancy afterward without genetic testing.

This study was approved by the Ethics Board of School of Life Sciences (No. 2021-1-12), Central South University, in accordance with the approved ethical guidelines. Informed consent was obtained from the patient of this family. The patient was not involved in the development of the research, and there were no core outcome sets used in this study.

### 2.2 Karyotype analysis and copy number variation sequencing

Karyotype analysis was conducted by G-banding where the metaphase peripheral blood of the proband was prepared using standard procedures (McGowan-Jordan et al., 2016), and a level of 550 bands was carried out using these metaphase spreads. At least 50 metaphase spreads were examined.

Genome-wide CNV-seq of the proband was performed using a chromosome copy number variation detection kit (Berry Genomics, Hangzhou, Zhejiang, China). After library construction and purification, library sequencing was performed on a NextSeq 500 sequencer (Illumina, San Diego, CA, United States). Data analysis was performed by chromosome copy number variation data analysis software V3 (Berry Genomics, Beijing, China).

### 2.3 Whole-exome sequencing

DNA was extracted following peripheral blood and stored for genetic testing. WES in the family trio was performed using stored DNA. The exomes were hybridized and captured by the xGen^®^ Exome Panel v1.0 (Integrated DNA Technologies, Coralville, Iowa, United States), and 100-bp paired-end reads were generated on a HiSeq 2000 system (Illumina, San Diego, CA, United States). Data analysis was performed according to the protocols described previously (Luo et al., 2019), and potential disease-causing variants were confirmed by following Sanger sequencing.

### 2.4 RNA extraction

The TRIzol reagent (Invitrogen, Carlsbad, CA, United States) was used following the manufacturer’s instructions to extract the total RNA from peripheral blood lymphocyte cells of the family members and healthy control individuals, including two males (21 and 34 years old), two females (26 and 28 years old), and two boys (8 and 11 years old). The isolated RNA was reverse-transcribed into cDNA using a Revert Aid First Strand cDNA Synthesis Kit (Thermo Fisher Scientific, Carlsbad, CA, United States) for the following real-time quantitative PCR and reverse transcription PCR experiments.

### 2.5 Real-time quantitative PCR and reverse transcription PCR

Real-time quantitative PCR (qPCR) assay was carried out on the LightCycler platform (Roche, Boston, MA, United States) with the Maxima SYBR^®^ Green Master Mix (Thermo Fisher Scientific, Carlsbad, CA, United States). The forward primer of *CEP152* was 5′-AAC​TTC​GTG​GGC​AGT​ACA​TT-3′, and the reverse primer was 5′-TTC​GGG​CGG​TTT​CTT​GAC-3’. Relative gene expression levels were determined using the 2^-ΔΔCt^ method after normalizing the mRNA level of *UBE2D2* amplified by the primer pair of 5′-GAT​CAC​AGT​GGT​CTC​CAG​CA-3′ and 5′-CGA​GCA​ATC​TCA​GGC​ACT​AA-3’. Three replicates were analyzed.

Reverse transcription PCR (RT-PCR) was performed by the forward primer (5′-ATT​TGG​ATG​CCA​CAG​TCA​C-3′) in exon 9 and the reverse primer (5′-TGG​TGC​TGC​TGA​TAA​GTC​C-3′) in exon 16. Agarose gel electrophoresis was used to ascertain the effect of the novel *CEP152* non-classical splice-site variant on the mRNA splicing pattern. After gel electrophoresis, PCR products were purified by excision and purification of the bands, and then sent for Sanger sequencing.

## 3 Results

### 3.1 Clinical characteristics

Physical examination: The 7-year-old boy (the proband) presented with microcephaly (head circumference 31 cm, <–3SD, and with bird-headed profile), short stature, slanting forehead, high bridge of the nose, beak nose, and micrognathia (Figure 1B). His height was 93 cm (<-3SD) and weight was 12 kg (<-3SD) (Figure 1C). He had multiple oral caries, pigeon toes, and the fifth finger clinodactyly. The detailed phenotypes are shown in Table 1.

**TABLE 1:** Clinical findings in the SCKL5 proband described in this study.

Characteristic	II-1
Sex	Male
Age (years)	7
*CEP152* variants	c.1060C>T/c.1414-14A>G
Growth	
Head circumference (cm)	31
Height (cm)	93
Weight (kg)	12
Facial and neck	
Head	
Microcephaly	+
Face	
Sloping forehead	+
Micrognathia	+
Retrognathia	+
Eyes	
Downslanting palpebral fissures	N.A.
Strabismus	N.A.
Blepharophimosis	+
Laryngotrachea	
Laryngotracheal malacia	+
Endobronchial inflammation	+
Nose	
Beaked nose	+
High nasal bridge	+
Mouth	
High-arched palate	N.A.
Teeth	
Hypodontia	−
Oligodontia	−
Enamel hypoplasia	−
Selective tooth agenesis	+
Chest	
11 pairs of ribs	N.A.
Genitourinary	
Cryptorchidism	Cryptorchidism
Skeletal findings	
Delayed bone age	N.A.
Fifth finger clinodactyly	+
Pes planus	+
Neurologic	
Simplified gyri	−
Intellectual disability	+

N.A. not applicable/unknown; accession number *CEP152*: NM_014985.4.

Intelligence test: The patients were tested with the Chinese Wechsler Intelligence Scale for Children (C-WISC) and scored 57, 68, and 59 on the verbal intelligence quotient (VIQ), performance intelligence quotient (PIQ), and full-scale intelligence quotient (FIQ), respectively, which suggested intellectual disability.

Auxiliary examination: Brain magnetic resonance imaging (MRI) indicated no obvious abnormality (Figure 1D). The levels of amino acid and acylcarnitine spectrum analysis were in the normal ranges (Supplementary Figure S1). Fiberoptic bronchoscope examination showed that the proband had laryngotracheal malacia and endobronchial inflammation (Supplementary Figure S2). Electroencephalogram (EEG) and brain electrical activity mapping (BEAM) were recorded during sleep. The background activity of the EEG in the proband mainly comprised intermittent rhythmic *d* and θ activities within each brain region. These waveforms were irregular with sporadic low-amplitude β-rhythm (<10 μV). The results showed no obvious abnormality in EEG during sleep, and no seizure wave was found in the whole range.

### 3.2 Identification of two novel variants in *CEP152*

**FIGURE 2:**
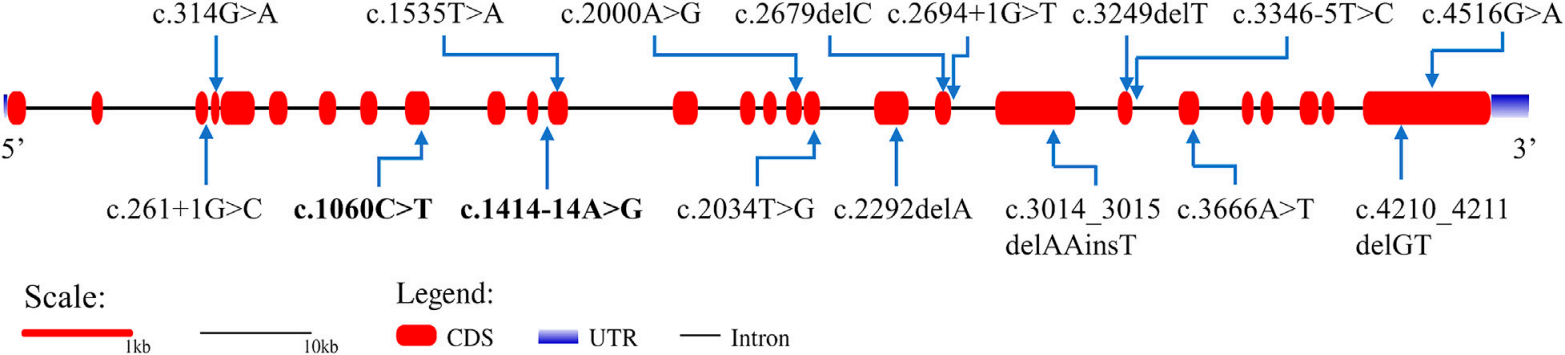
Gene structure of *CEP152* and the distribution of variants that have been reported to cause SCKL. Bold variants are those identified in this study. The red boxes represent the coding sequence (CDS). The blue gradient boxes represent the 5′ and 3′ untranslated regions (UTR).

G-banding karyotype analysis of the proband revealed a normal male karyotype of 46, XY. The genome-wide CNV-seq analysis showed that no chromosomal aneuploidy or known genome copy number variation above 100 kb was detected. We identified a novel compound heterozygous variant in *CEP152* by using trio-WES, which was composed of a paternally inherited c.1414-14A>G splice-site variant in intron 12 and a maternally inherited c.1060C>T (p.Arg354*) non-sense variant in exon 9 (Supplementary Figure S3A). The two variants have not been reported previously in public databases. The online software application speculated that the non-sense variant led to the loss of nearly four-fifths of the CEP152 protein and was considered to be damaging (http://varcards.biols.ac.cn/). According to the standards and guidelines for the interpretation of sequence variants proposed by the American College of Medical Genetics and Genomics (ACMG) and the Association for Molecular Pathology (AMP) (Richards et al., 2015), the variant was evaluated as “likely pathogenic”. Since the splice-site variant (c.1414-14A>G) detected in our study was not in the 1–2 nucleotide site near the junction of an exon and intron, the variant was evaluated as “uncertain significance (VUS)” (Supplementary Figures S3B, C). The positions of these two variants are shown in Figure 2.

### 3.3 Total *CEP152* mRNA expression analysis and conformation for skipping exon 12

**FIGURE 3:**
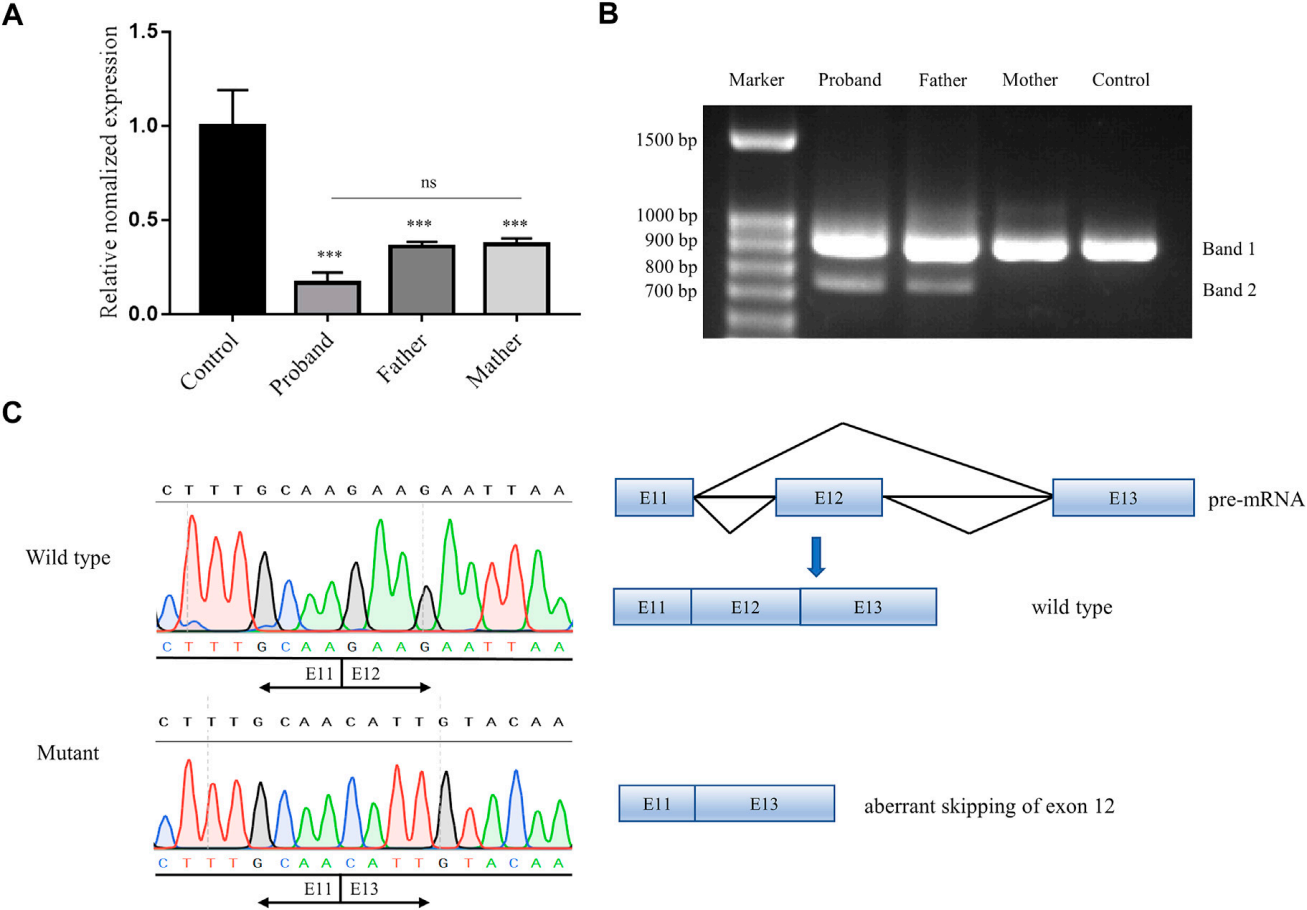
Pathogenicity verification of a novel splice-site variant in our study. **(A)** qPCR result showed the relative expression of *CEP152* of the proband and his parents to be 16.6%, 36.0%, and 37.3%, respectively. ***p < 0.001 (*t*-test). **(B)** Agarose gel electrophoresis showed that spanning exon amplification products in the proband and his father, who also carried the splice-site variant, were compared with his mother and the healthy control individual. All subjects displayed a common 907-bp band (band 1, wild type), while the proband and his father had an additional 743-bp band (band 2, mutant). **(C)** Left half is Sanger sequencing showing the 743-bp band deleted exon 12 (164 bp nucleotides of *CEP152* cDNA) compared to the 907-bp band (matching to the reference sequence). The right half is the schematic representation of the normal and aberrant transcripts.

To further evaluate the potential effect of the non-classical splice-site variant c.1414-14A>G of *CEP152*, qPCR and RT-PCR were performed. The qPCR revealed that the expression level of *CEP152* decreased drastically (proband: 83.4%, father: 64.0%, and mother: 62.7%) in the family compared to a healthy control individual (Figure 3A). Moreover, splicing patterns of the proband and his parents were analyzed by spanning exon amplification, agarose gel electrophoresis, and Sanger sequencing. In the lanes of the proband and his father, an additional 743 bp amplification product was observed, which is absent in his mother and the healthy control individual (Figure 3B). The left half of Figure 3C displayed a single exon 12 skipping, including 164 bp nucleotides, that was found in the 743-bp PCR product by Sanger sequencing, which led to the c.1414_1577del (p.Glu472_Ser526delfs*9) frameshift variant of *CEP152*. The right half showed the schematic representation of the normal and aberrant transcripts, respectively. Thus, our study further demonstrated the abnormality of the splicing pattern.

## 4 Discussion

SCKL has been mainly classified into nine subtypes caused by different genes (Table 2). Biallelic pathogenic variants in *CEP152* have been mainly described in patients with autosomal recessive primary microcephaly-9 (MIM#614852, MCPH9) and Seckel syndrome 5. Based on the Human Gene Mutation Database (HGMD), 32 variants in *CEP152* have been identified in patients and have reported to be associated with different kinds of diseases including microcephaly, Seckel syndrome, atrioventricular septum defect, and autism spectrum disorder. Nevertheless, the pathogenicity of some variants is still unknown. So far, only 14 variants involved in six literature studies were reported to cause SCKL 5 (Figure 2; Supplementary Table S1).

**TABLE 2:** Correlation between genes and subtypes of SCKL.

Subtype	Gene(s)	Location of the chromosome
SCKL1	*ATR* (MIM 601215)	3q23
SCKL2	*RBBP8* (MIM 604124)	18q11
SCKL4	*CENPJ* (MIM 609279)	13q12
SCKL5	*CEP152* (MIM 613529)	15q21
SCKL6	*CEP63* (MIM 614724)	3q22
SCKL7	*NIN* (MIM 608684)	14q22
SCKL8	*DNA2* (MIM 601810)	10q21
SCKL9	*TRAIP* (MIM 605958)	3p21
SCKL10	*NSMCE2* (MIM617246)	8q24
Unassorted SCKL	*CDK5RAP2* (MIM 608201)	9q33
	*PLK4* (MIM 605031)	4q28

The report of a Seckel syndrome locus on chromosome 14q, designated SCKL3, by Kilinc et al. (2003) was found to be in error.

Although all the cases, including the case in our study, reported proportionate short stature, disproportionate head circumference in growth, sloping forehead, micrognathia, beaked nose in facial dysmorphism, and intellectual disability, some phenotypic heterogeneity was also displayed among different populations and pathogenic variants. For example, four Turkish patients with homozygous c.261 + 1G>C pathogenic variants in *CEP152* showed high-arched palate, selective tooth agenesis, enamel hypoplasia, fifth finger clinodactyly, genital anomalies, and simplified gyri. A French individual with the same genotype, however, did not manifest such phenotypes (Kalay et al., 2011). Furthermore, different variants may associate with various other exceptional phenotypes. Compound heterozygous c.1535T>A and c.3346-5T>C results in the additional phenotype of the cleft palate (Hong et al., 2019), while c.2000 A>G/c.4210-4211delGT leads to scoliosis (Kalay et al., 2011). In our case, laryngotracheal malacia and endobronchial inflammation have not been reported previously. Since CEP152 is necessary for centrosome duplication (Kodani et al., 2015), centriole formation (Cizmecioglu et al., 2010), and G2/M transition of the mitotic cell cycle, and has also been reported to interact with CDK5RAP2, WDR62, CEP63, CEP131, PLK4, and CENPJ (Sir et al., 2011), phenotypic heterogeneity in patients with SCKL may be due to diverse proteins affected by specific variants of *CEP152*. Therefore, the correlation between these phenotypes and the location of the variants in *CEP152* along with the associated pathways needs to be further verified by more case analysis and functional studies.

Meanwhile, since the offspring of an affected family has a 25% chance of recurrence risk, it is important for the family of SCKL to undertake genetic testing, counseling, and prenatal diagnosis for the subsequent pregnancy (Gupta et al., 2014; Akkurt et al., 2019). In 2019, Capalbo et al. found four deleterious variants in *CEP152* detected by exome sequencing (ES) in 14,125 gamete donors and couples with no genetic diseases in their family history who underwent *in vitro* fertilization (IVF) treatment, suggesting the carrying rate of the pathogenic variant in *CEP152* can be as high as 0.28% (Capalbo et al., 2019). With advances in next-generation sequencing and decreasing costs of this technology, more and more gene panels are applied to preconception/prenatal carrier screening. Considering the severity and high carrier rate of the SCKL, it is necessary to include SCKL-related genes in the gene list for carrier screening. Once screening shows that both of the couple carry the pathogenic variants in the same gene, preimplantation genetic testing for monogenic disorder (PGT-M) or prenatal diagnosis can be used to provide a basis for the selection and clinical management of the couple’s pregnancy. In this study, we recruited a family with suspected SCKL and collected their history of adverse pregnancy. The proband had typical SCKL phenotypes: a typical ‘bird-head’ facial appearance, severe microcephaly, short stature, intellectual disability, dysphasia, caries, and fifth finger clinodactyly. Unfortunately, due to the difficulty in obtaining detailed ultrasound information on the affected fetus, the analysis of the relationship between the genotype and ultrasound phenotype could not be conducted. Here, we present genetic evidence by trio-WES where biallelic pathogenic variants in *CEP152* causing Seckel syndrome 5 were detected. In addition, the pathogenicity of the c.1414-14A>G variant was upgraded from VUS to pathogenic by adding functional evidence (applicable to PVS1 null variant interpretation of the ACMG guideline) of single exon 12 skipping, a frameshift variant of the c.1414_1577del (p.Glu472_Ser526delfs*9) of *CEP152*, which caused the exposure of premature termination codons (PTCs), and was predicted to be degraded by the non-sense-mediated mRNA decay (NMD) pathway because of locating PTC at more than 50–55 nucleotides upstream of the exon–exon junction triggering efficient NMD (Abou Tayoun et al., 2018; Dyle et al., 2020). It was worth noting that the splice-site variant was not located in the classical ±1 or ±2 position, and further splicing analysis confirmed its pathogenicity. All these results provided new insights into splice-site variants and a basis for genetic counseling for the couple’s subsequent pregnancy.

In conclusion, our study identified two novel variants, c.1060C>T and c.1414-14A>G, in a compound heterozygotic form of *CEP152* and proved them to be the likely pathogenic factor for the phenotype of the affected family. The non-classical splice-site variant was confirmed to reduce the mRNA expression level and alter the splicing pattern by qPCR and RT-PCR, respectively. These findings expand the pathogenic variant spectrum of *CEP152* and provide more information for understanding SCKL and give evidence for the genetic and reproductive counseling of the family.

## Data Availability

The data presented in the study are deposited in the open database Genome Sequence Archive (Chen et al., 2021) in National Genomics Data Center (CNCB-NGDC Members and Partners, 2022), and the accession number is HRA003542.
