# Supplementary material for: Two novel variants in CEP152 caused Seckel syndrome 5 in a Chinese family
Source: Front Genet. 2023 Jan 4;13:1052915. doi: 10.3389/fgene.2022.1052915 (PMC9845263; doi:10.3389/fgene.2022.1052915)
Supplement: Supplementary file 1 [file DataSheet1.doc]

**Table S1.** Genetic variants included in the Human Gene Mutation Database (HGMD) and this study.

| Gene | cDNA | AA change | MAF | Disease in HGMD | Reference |
| --- | --- | --- | --- | --- | --- |
| *CEP152*  (NM_ 014985.4) | c.95A>C | p.Gln32Pro | N.A. | Microcephaly, congenital | Pmid: 30214701 |
|  | c.121A>G | p.Met41Val | N.A. | Microcephaly | Pmid: 32369273 |
|  | c.255T>G | p.Ser85Arg | 0.0002 | Atrioventricular septum defect, partial | Pmid:25996639 |
|  | c.261+1G>C | - | N.A. | Seckel syndrome | Pmid:21131973 |
|  | c.314G>A | p.Trp105* | N.A. | Seckel syndrome | Pmid:30609409 |
|  | c.343C>T | p.Arg115* | N.A. | Epileptic encephalopathy? | Pmid:31322791 |
|  | c.794A>C | p.Gln265Pro | N.A. | Microcephaly, Primary | Pmid:20598275 |
|  | **c.1060C>T** | **p.Arg354*** | **N.A.** | **Seckel syndrome** | **This study** |
|  | c.1180A>G | p.Ile394 Val | 0.0021 | Atrioventricular septum defect, partial | Pmid:25996639 |
|  | **c.1414-14A>G** | - | **N.A.** | **Seckel syndrome** | **This study** |
|  | c.1535T>A | p.Leu512* | N.A. | Seckel syndrome | Pmid:31055814 |
|  | c.1718A>C | p.Asp573Ala | N.A. | Autism spectrum disorder | Pmid:28720891 |
|  | c.1741C>A | p.Leu581Ile | N.A. | Atrioventricular septum defect, complete | Pmid:25996639 |
|  | c.2000A>G | p.Lys667Arg | 0.0038 | Seckel syndrome | Pmid:21131973 |
|  | c.2034T>G | p.Tyr678* | 0.0005 | Seckel syndrome | Pmid:21131973 |
|  | c.2148-17G>A | - | N.A. | Microcephaly, Primary | Pmid:28454995 |
|  | c.2292delA | p.Lys764Asnfs*18 | N.A. | Seckel syndrome | Pmid:31980526 |
|  | c.2668C>T | p.Gln890* | N.A. | Microcephaly | Pmid:32369273 |
|  | c.2694+1G>T | - | N.A. | Seckel syndrome | Pmid:21131973 |
|  | c.2679delC | p.Ser894Leufs*2 | N.A. | Seckel syndrome | Pmid:31589614 |
|  | c.2878T>C | p.Trp960 Arg | 0.0032 | Atrioventricular septum defect | Pmid:25996639 |
|  | c.2959C>T | p.Arg987* | 3.234e-5 | Primary autosomal recessive microcephaly 9 | Pmid:20598275 |
|  | c.3014_3015delAAinsT | p.Lys1005Ilefs*16 | N.A. | Seckel syndrome | Pmid:26436113 |
|  | c.3065A>G | p.Gln1022Arg | N.A. | Autism spectrum disorder | Pmid:28831199 |
|  | c.3149T>C | p.Leu1050Pro | N.A. | Primary autosomal recessive microcephaly 9 | Pmid:22775483 |
|  | c.3249delT | p.Val1084Cysfs*7 | 0.0000481 | Seckel syndrome | Pmid:31589614 |
|  | c.3313C>G | p.Leu1105Val | 0.0021 | Atrioventricular septum defect, partial | Pmid:25996639 |
|  | c.3346-5T>C | - | N.A. | Seckel syndrome | Pmid:31055814 |
|  | c.3508_3510delAAC | p.Asn1170del | N.A. | Primary autosomal recessive microcephaly 9 | Pmid:22775483 |
|  | c.3666A>T | p.Lys1222Asn | 0.000012 | Seckel syndrome | Pmid:31589614 |
|  | c.4210_4211delGT | p.Val1404Phefs*3 | N.A. | Seckel syndrome | Pmid:21131973 |
|  | c.4516G>A | p.Val1506Ile | 0.000112 | Seckel syndrome | Pmid:31589614 |
|  | c.4570A>G | p.Thr1524Ala | 0.0005 | Atrioventricular septum defect, complete | Pmid:25996639 |
|  | ~10.56 Mb, adjacent to gene (described at cDNA level) | | N.A | Intellectual disability and hypoplasia of corpus callosum ? | Pmid: 25315429 |

AA: Amino acid; N.A.: Not applicable/unknown; MAF: Minor Allele Frequency.


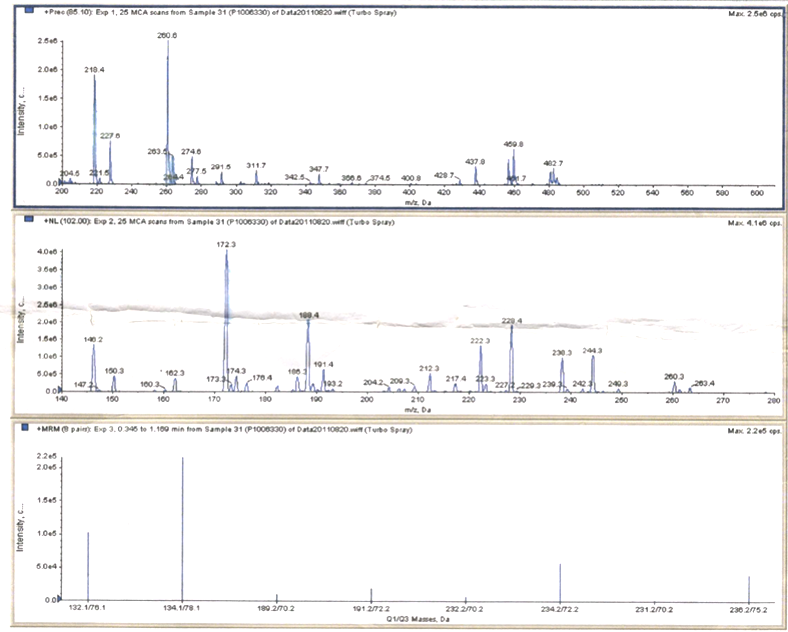


**Figure S1.** Analysis report of amino acid and acylcarnitine spectrum of genetic metabolic diseases. An examination of Serum amino acid and acylcarnitine profiles showed no apparent abnormalities.


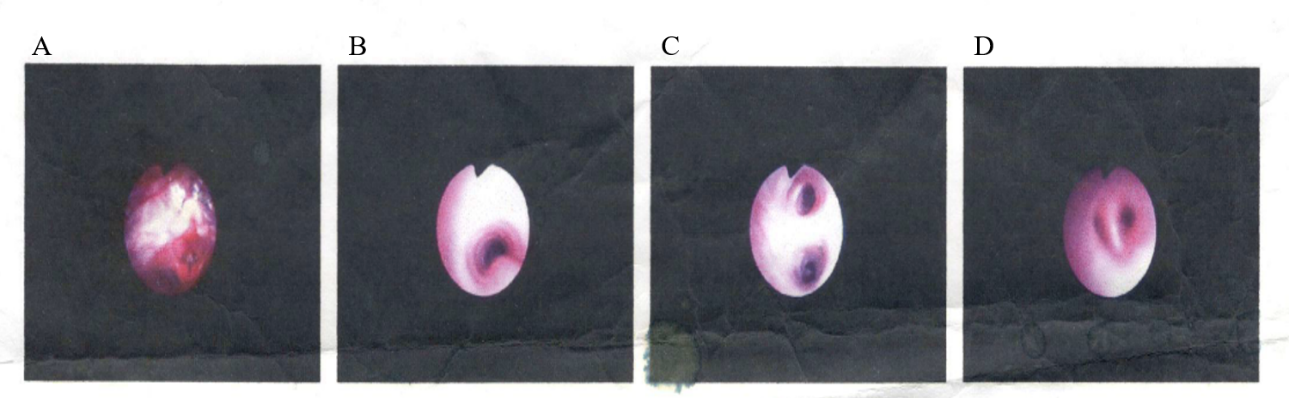


**Figure S2.** The result of fibero-bronchoscope examination. The results showed that the proband had laryngotracheal malacia and endobronchial inflammation. (A) Epiglottis. (B) Trachea. (C) Left superior and inferior lobar bronchus. (D) Superior lobe of right lung.


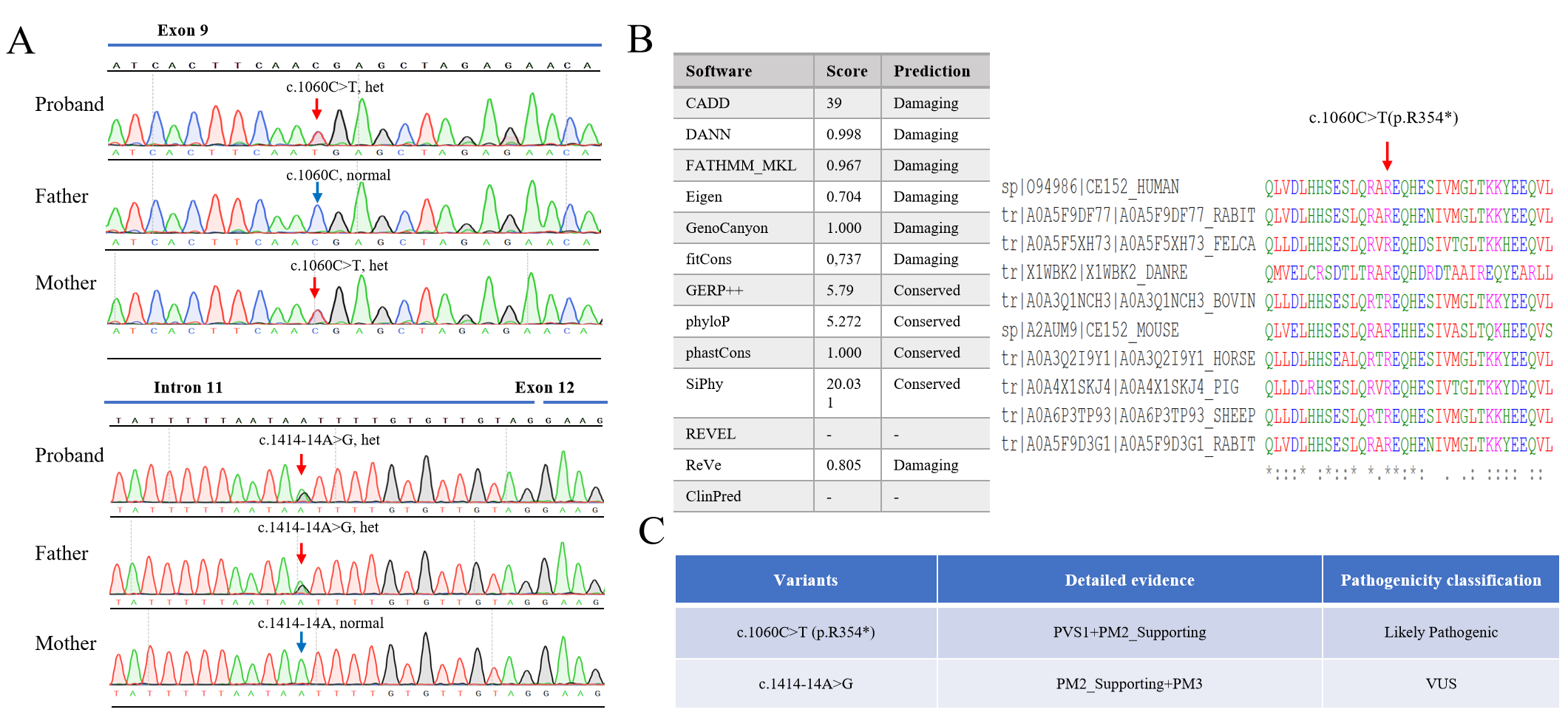


**Figure S3.** Molecular findings from sequencing and software prediction. **(A)** Sanger sequencing showed compound heterozygous mutations (c.1060C>T, c.1414-14A>G, NM_014985.3) in *CEP152* in the proband, the nonsense variant c.1060C>T inherited from his mother, and the non-canonical splice site variant c.1414-14A>G inherited from his father respectively. **(B)** Online software predicted that the nonsense mutation in our study was damaging. **(C)** The nonsense variant (c.1060C>T) and the splice site variant (c.1414-14A>G) are classified as likely pathogenic variant and uncertain significance variant (VUS) respectively, according to the guideline of ACMG/AMP.
